# Absence of “Cytokine Storm” in Hospitalized COVID-19 Patients: A Retrospective Cohort Study

**DOI:** 10.3390/idr13020036

**Published:** 2021-04-19

**Authors:** Maeghan L. Ciampa, Thomas A. O’Hara, Constance L. Joel, Melinda M. Gleaton, Kirti K. Tiwari, Daniel M. Boudreaux, Balakrishna M. Prasad

**Affiliations:** 1 Department of General Surgery, Dwight D Eisenhower Army Medical Center, Fort Gordon, GA 30905, USA; maeghan.l.ciampa.mil@mail.mil (M.L.C.); thomas.a.ohara7.mil@mail.mil (T.A.O.); constance.l.joel.mil@mail.mil (C.L.J.); 2 Department of Pathology, Dwight D Eisenhower Army Medical Center, Fort Gordon, GA 30905, USA; melinda.m.gleaton.civ@mail.mil; 3 Department of Clinical Investigation, Dwight D Eisenhower Army Medical Center, Fort Gordon, GA 30905, USA; kirti.k.tiwari.mil@mail.mil (K.K.T.); daniel.m.boudreaux.mil@mail.mil (D.M.B.)

**Keywords:** coronavirus, immunosuppressant, dexamethasone, interleukin, COVID-19, cytokine

## Abstract

**Background:** A rapidly growing number of publications cite “cytokine storm” as a contributing factor in coronavirus disease 2019 (COVID-19) pathology. However, a few recent reports led to questioning of “cytokine storm” theory in COVID-19. This study’s primary goal is to determine if exaggerated cytokine response in the range of a “cytokine storm” develops during the initial weeks of hospitalization in COVID-19 patients. **Methods:** Five proinflammatory cytokines reported to be involved in “cytokine storm” and elevated in COVID-19 (IL-6, IL-8, TNF-α, MCP-1, and IP-10) were analyzed in COVID-19, influenza (with “cytokine storm”: CS), and burn injury patients. The effect of dexamethasone use on cytokine response in COVID-19 was also analyzed. **Results:** None of the five cytokines in COVID-19 patients reached the lower threshold (95% CI) of the influenza (CS) group at any point during the study period. Furthermore, mean concentrations of all five cytokines in the influenza (CS) group and IL-6, IL-8, TNF-α in the burn group were significantly greater than in COVID-19 patients (*p* < 0.01). Dexamethasone treatment did not significantly alter the concentrations of any of the cytokines analyzed. **Conclusions**: Exaggerated cytokine response similar to “cytokine storm” was not observed in COVID-19 patients during two weeks of hospitalization.

## 1. Introduction

Coronavirus disease 2019 (COVID-19), which was declared a pandemic by the World Health Organization, is caused by severe acute respiratory syndrome coronavirus 2 (SARS-CoV-2). Pharmacotherapeutic choices for COVID-19 depend on the severity of symptoms, time after infection, patient characteristics and comorbidities [1,2]. Although no specific cure is available at present, interventions commonly being used include antiviral, anticoagulant and immunosuppressant drugs. The therapeutic standard of care has evolved along with our understanding of the pathophysiology of COVID-19 over the past year.

The rationale for using antiviral drugs is obvious, and remdesivir is the first COVID-19 treatment to be approved by the FDA in October 2020 [3]. Intracellular replication of SARS-CoV-2 depends on RNA-dependent RNA polymerase, and remdesivir can decrease the viral load by inhibiting this enzyme. The presence of disseminated intravascular coagulation and venous thromboembolism justified anticoagulant use in COVID-19 patients [4]. COVID-19 patients were reported to have hyperimmune reactions based on elevated circulating cytokine concentrations and altered lymphocyte profile. Indeed, one of the first characterizations of COVID-19 clinical phenotype reported elevated concentrations of inflammatory cytokines [5]. Furthermore, some cytokines’ plasma concentrations were associated with disease progression and severity [6,7,8]. These studies led to the widely publicized theory of “cytokine storm” in COVID-19 and even drew parallels with cytokine release syndrome and secondary hemophagocytic lymphohistiocytosis [9,10]. 

However, critical quantitative analysis of COVID-19 cytokine data than hyperinflammatory conditions led to questioning of the “cytokine storm” theory of COVID-19 [11]. A recent study showed that plasma concentrations of three proinflammatory cytokines are not as elevated in COVID-19 as in sepsis with acute respiratory distress syndrome [12]. Almost all of the studies evaluated cytokine profiles in hospitalized COVID-19 patients within 48 h of admission. A cytokine storm, particularly in the context of an active infection, may take several days to manifest [13]. Thus, this study’s primary goal is to test the hypothesis that an exaggerated cytokine response may develop during the initial weeks of hospitalization in COVID-19 patients. In addition, we tested the effect of dexamethasone treatment on cytokine concentrations and analyzed if its use is responsible for masking the presence of a “cytokine storm”.

## 2. Methods

All study procedures were performed in compliance with Eisenhower Army Medical Center institutional review board. Daily blood samples were collected from COVID-19 PCR-positive patients admitted to the hospital between March and October 2020. Leftover serum or plasma samples after clinical pathology tests were stored at −80 °C. Cytokine concentrations were measured using a 46-plex magnetic bead assay according to the manufacturer’s instructions (EMD-Millipore, Burlington, MA, USA). Samples in our tissue bank from influenza, burn or control patients (obtained before 2020) were also analyzed in the same assays. Although there is no established quantitative criterion for “cytokine storm”, several proinflammatory cytokines are increased by orders of magnitude during cytokine release syndrome with IL-6 concentrations in the range of 10 ng/mL [14]. We had previously measured IL-6 in several respiratory infections and used a cohort of influenza with pneumonia (ICD J11.0) as an appropriate positive control for “cytokine storm” in a viral infection. IL-6 concentration greater than 1 ng/mL was used to identify influenza (CS) samples. Samples from patients with greater than 40% total body surface area burns and control patients were used as additional references representing trauma-induced and normal cytokine concentrations, respectively. Relative abundance of antibodies against the receptor-binding domain (RBD) of COVID-19 spike protein was also measured using a SARS-CoV-2 multi-antigen IgG assay kit (Luminex, Austin, TX, USA).

Clinical parameters, including dexamethasone use and patient characteristics, were obtained from retrospective chart reviews. Cytokine concentrations of COVID-19 patients were analyzed in samples collected on alternate days, beginning with the day of hospitalization. Analysis was performed in samples up to 2 weeks in patients that received dexamethasone (6 mg/day for 10 days) and up to 1 week in those that did not. Sample inclusion depended on their availability and length of the patient’s hospital stay. One patient in the dexamethasone group received tocilizumab on day 4 after hospitalization. All post-tocilizumab samples in this patient were excluded from analysis because of a drug-induced increase in IL-6.

Five of the 46 cytokines measured were included in the data analyses. Interleukin-6 (IL-6), IL-8, tumor necrosis factor-α (TNF-α), monocyte chemoattractant protein-1 (MCP-1), interferon γ-induced protein-10 (IP-10) are typical proinflammatory cytokines whose concentrations were reported to be elevated in cytokine release syndrome and in COVID-19 [5,6,14]. In addition, IL-6, IL-8 and IP-10 concentrations were correlated with COVID-19 progression and severity [6,7]. All data points for each COVID-19 patient across the monitoring period were averaged. Geometric means of COVID-19 patients with dexamethasone treatment (DEX) and those with no-dexamethasone treatment (no-DEX) were compared to influenza (CS), burn and control groups. Log-transformed data were analyzed with ANOVA followed by Tukey’s multiple comparisons test. To further assess dexamethasone’s effects, the data points while a patient is on dexamethasone treatment were compared to those before and after the treatment using paired *t*-tests. Paired *t*-tests on log-transformed values were used to assess changes in cytokine concentrations throughout hospitalization. Age and BMI comparisons were made with *t*-test, while Fisher’s exact test was used for all other patient characteristics. A *p* value of < 0.01 was used to define significance in all statistical comparisons.

## 3. Results

Clinical data of hospitalized COVID-19 patients with or without dexamethasone treatment are shown in Table 1. The two groups had comparable patient characteristics except hypertension incidence rates.

**Figure 1:**
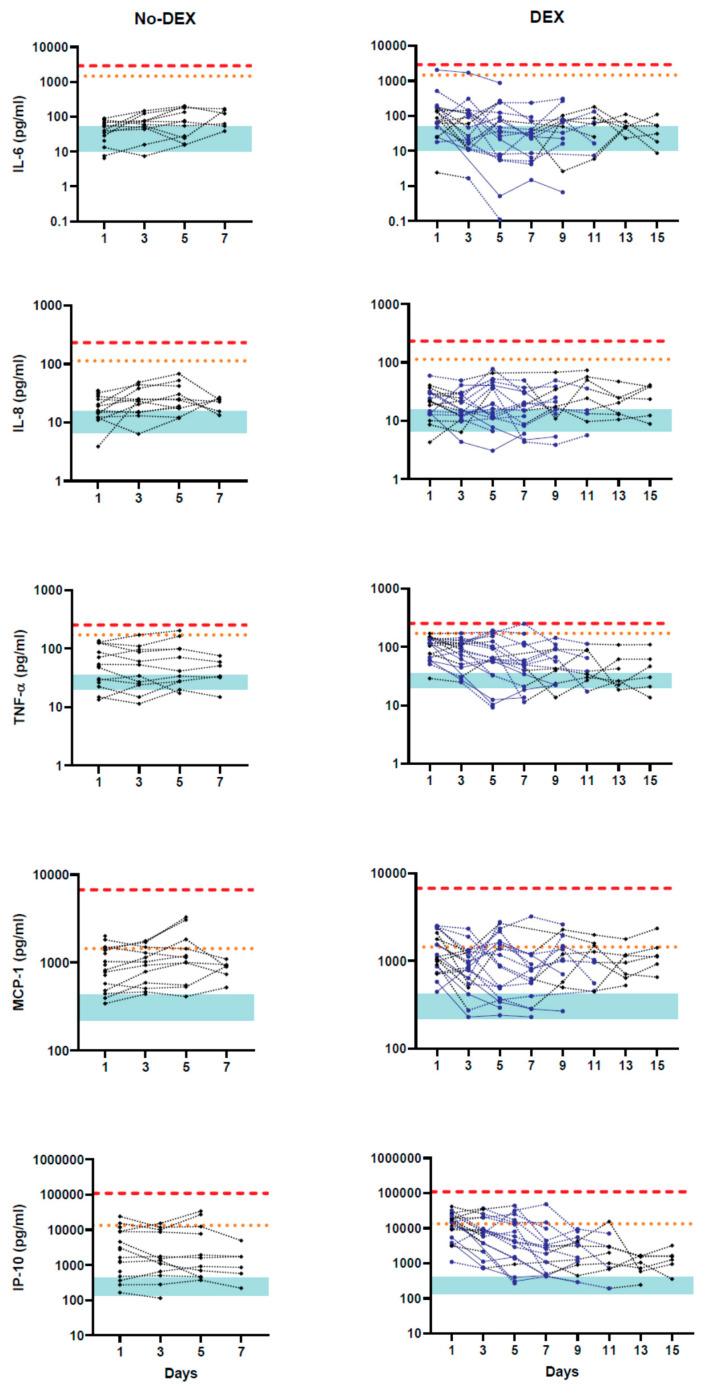
Cytokine concentrations in COVID-19 patients after hospitalization. Circulating cytokine concentrations for each patient are shown on a logarithmic scale. The left panel shows patients in the no-dexamethasone treatment (DEX) group. In the DEX group, data points and lines when the patient is on dexamethasone are shown in blue, while those before and after treatment are shown in black. Four patients were on dexamethasone treatment throughout the observation period, and their data points are connected with a solid blue line. The red dashed line shows the lower 95% confidence limit of influenza (CS) patient values, while the dotted line represents the lower limit for burn injury patients. Upper and lower confidence limits for the control group are shown in turquoise shaded areas.

Circulating cytokine concentrations on alternate days after hospitalization in each patient are shown in Figure 1. None of the five cytokine values reached the lower threshold (95% CI) of the influenza (CS) group at any point during the study period in DEX or no-DEX group. Furthermore, mean concentrations of all five cytokines in COVID-19 were significantly lower than in the influenza (CS) group (Table 2). IL-6, IL-8 and TNF-α concentrations were significantly lower in COVID-19 groups compared to burn patients. TNF-α, MCP-1 and IP-10 (but not IL-6 or IL-8) were significantly higher in both COVID-19 groups than the control group. However, the magnitude of differences for these three cytokines between normal and COVID-19 groups was substantially lower than the differences between COVID-19 and influenza (CS) groups. Concentrations of all five cytokines were similar in DEX and no-DEX groups (*p* > 0.01). Several other cytokines and chemokines showed similar circulating concentrations in COVID-19 patients. Either modest (but not in the “cytokine storm” range) or no increase in concentrations compared to the control group were observed (Supplemental Table S1).

**Figure 2:**
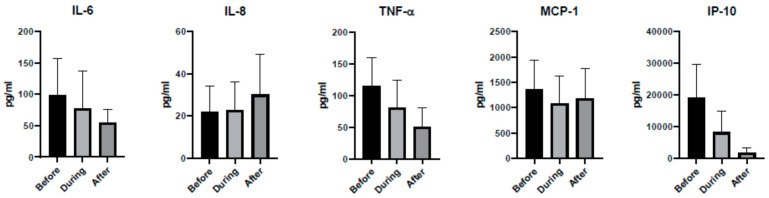
Effect of dexamethasone on cytokine concentrations in COVID-19 patients. Average cytokine values before, during and after dexamethasone treatment are shown as mean with standard deviation. Paired t-tests showed no statistical difference between before and during dexamethasone treatment for any of the cytokines (*p* > 0.01, n = 10). Similarly, no significant difference was observed between during and after periods (*p* > 0.01, n = 8).

To further assess dexamethasone’s effect on cytokine concentrations, a pair-wise comparison was done within each subject before, during and after treatment. Figure 2 shows that none of the cytokine concentrations were statistically different between before and during treatment periods (*p* > 0.01). Similarly, no statistically significant differences were observed between during and after treatment periods.

**Figure 3:**
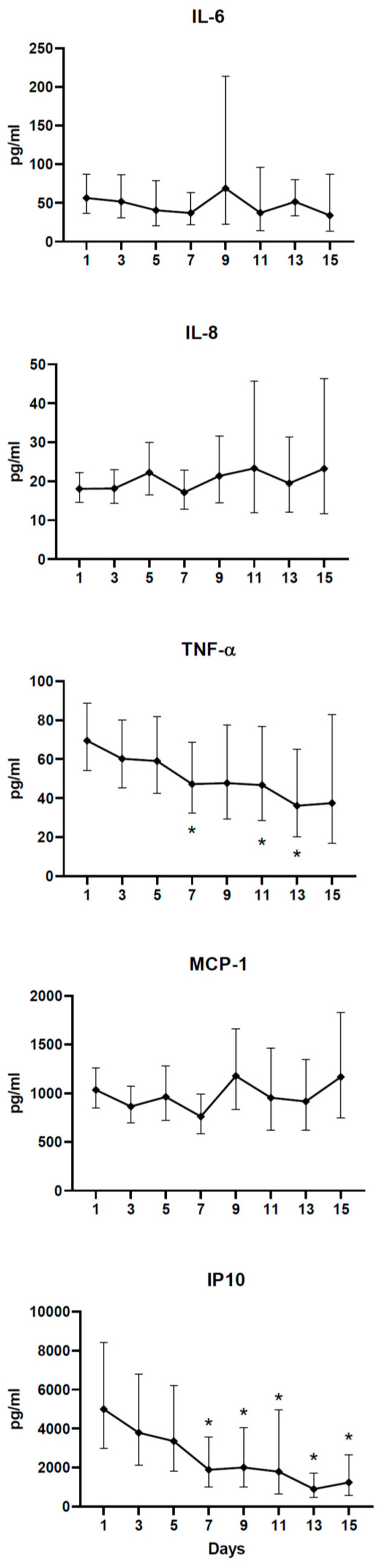
Time-course of cytokine response in COVID-19 patients after hospitalization. Cytokine concentrations of all patients in the study are shown as geometric mean with 95% CI. * indicates significant difference from day 1 values using paired *t*-test (*p* < 0.01).

We next analyzed the effect of time after hospitalization on cytokine concentrations. For this and all subsequent analyses, data from the DEX and no-DEX groups were combined, as there was no effect of dexamethasone treatment on cytokine concentrations. Figure 3 shows that TNF-α and IP-10 concentrations decreased significantly after 7 days of hospitalization. These data suggest that modest increases in cytokine concentrations are likely to decrease within two weeks, and the stage of infection is critical in the interpretation of cytokine responses.

**Figure 4:**
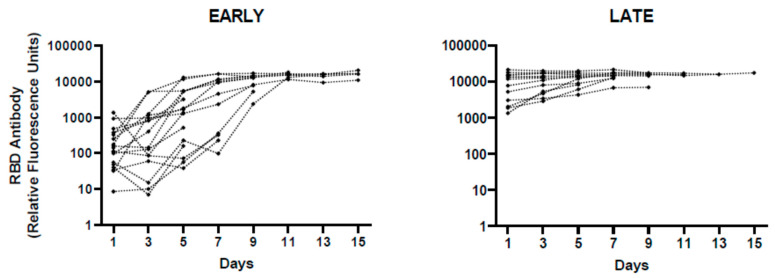
Receptor-binding domain (RBD) antibody response in COVID-19 patients. Relative RBD IgG concentrations in early and late infection groups are shown over the course of hospitalization. RBD value of 1100 on the first day of hospitalization was used to distinguish early and late stages of infections. N = 20 for early and 16 for late groups.

**Figure 5:**
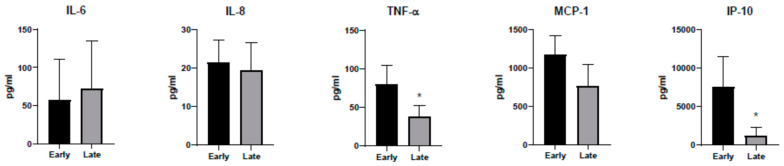
Effect of stage of infection on cytokine concentrations. Average cytokine values in early and late infection groups are shown as geometric mean with 95% CI. * represents a significant difference from the early group (*p* < 0.01). For monocyte chemoattractant protein-1 (MCP-1), the *p* value was 0.011.

To better assess the state of infection and to confirm appropriate adaptive immune response in our cohort, we measured the relative abundance of RBD antibodies in the same samples used for cytokine analysis. All patients developed robust RBD antibody responses by the seventh day of hospitalization regardless of dexamethasone treatment (Supplemental Figure S1). However, some patients showed maximal antibody response on the first day of hospitalization. Thus, we divided the patients into early and late infection groups based on RBD antibody abundance on the first day of hospitalization (Figure 4). Indeed, the average concentrations of TNF-α and IP-10 were significantly different between early and late infection groups (Figure 5). In addition, the concentrations of these two cytokines decreased with time, especially in the early infection group (Supplemental Figure S2). These data further illustrate that the modest increase in cytokines observed early during COVID-19 decreases within two weeks to baseline values.

## 4. Discussion

Exaggerated cytokine response, especially of the magnitude and pattern typically associated with “cytokine storm”, was not observed in COVID-19 patients during two weeks of hospitalization. Data presented above are the first direct comparison of cytokine concentrations in COVID-19 patients with “cytokine storm” samples using the same analysis platform. Furthermore, we addressed several confounding factors, such as assessing immunosuppressant use, stage of infection, time-dependent effects and used appropriate positive and negative controls. Dexamethasone treatment did not significantly alter concentrations of the cytokines analyzed in COVID-19 patients. Very modest increases in cytokine concentrations were observed even in early infection when antibody response was not well-developed. It is possible that hospitalized COVID-19 patients may be immunocompromised and are unable to mount a cytokine response. However, our antibody data shows that all patients developed robust antibody responses within a week of hospitalization and at least the adaptive immune system is adequately functioning. These findings, together with the use of appropriate control samples in the same analysis platform, enable us to conclude that “cytokine storm” is absent in hospitalized COVID-19 patients.

Elevated plasma cytokine concentrations have also been reported in SARS-1 and MERS patients [15,16]. Indeed, the magnitude and pattern of cytokine response to these earlier variants of coronavirus infections are similar to that in COVID-19. For example, maximum concentrations in pg/mL of IL-6 (<175), IL-8 (30), TNF-α (<20), MCP-1 (<300), IP-10 (<10,000) observed in SARS-1 [16] are similar to those measured in this study and are substantially lower than expected in a “cytokine storm”. Furthermore, these cytokines’ concentrations decreased over a 25-day post-SARS-1 infection period in patients with or without methylprednisolone treatment.

Given the similarities of cytokine concentrations reported among published reports on COVID-19, it is puzzling that “cytokine storm” theory has gained as much traction in scientific and medical communities. Several factors may have contributed to this mischaracterization. First, a statistically significant increase in cytokine concentrations was misconstrued as an exaggerated response without considering the relative magnitude of cytokine response [11]. Surrogate clinical (H-score, acute respiratory distress syndrome) and circulating biomarkers (Ferritin, CRP) were used as evidence for cytokine storm rather than a quantitative assessment of cytokines [9,17,18,19]. Finally, the rapid pace of research and publication process in this global pandemic may not have been conducive to critical data evaluation.

Immune response to viral infection serves the useful purpose of controlling the pathogen. However, exaggerated cytokine response observed during sepsis, chimeric antigen receptor-T cell therapy (CAR-T) or certain antibody therapies can cause undesirable adverse effects, including organ failure [20,21]. While the exact pattern and magnitude of exaggerated cytokine responses are not defined, common use of “cytokine storm” refers to observable clinical symptoms that can be ascribed to elevated cytokine concentrations. For example, elevated IL-6 concentrations are responsible for fever, hypotension, and neutropenia observed after CAR-T cell administration [22]. Biological effects of IL-6 are mediated by classical and trans-receptor signaling pathways, with the latter pathway primarily responsible for adverse effects of this cytokine. Trans signaling by soluble IL-6 receptors can only be achieved at very high circulating concentrations of IL-6 (ng/mL range) observed in septicemia and cytokine release syndromes [23]. The ability of IL-6 receptor antibodies (tocilizumab) to decrease fever and circulating concentrations of other proinflammatory cytokines demonstrates that elevated IL-6 in the ng/mL range is responsible for the adverse effects of “cytokine storm” [14,22]. IL-6 concentrations observed in COVID-19 patients are well below the levels expected to cause trans-signaling and are unlikely to contribute to any remote organ pathology. This information will help interpret the outcomes of ongoing clinical trials with tocilizumab in COVID-19 patients [24]. Two recent randomized, double-blind, placebo-controlled studies did not find the clinical benefit of tocilizumab [25,26] and reported IL-6 concentration was around 24 pg/mL in moderately ill COVID-19 patients [26]. A few studies that reported IL-6 values in the ng/mL range in some COVID-19 patients used different analysis platforms [27,28], underscoring the importance of using appropriate control groups and analytical techniques.

Dexamethasone decreased mortality and improved clinical outcomes in COVID-19 patients on mechanical ventilation or receiving supplemental oxygen [29]. On the contrary, a retrospective study did not find any benefit of using corticosteroids in critically ill COVID-19 patients [30]. In light of our findings, systemic immunosuppression can be ruled out as a contributing factor for dexamethasone’s utility. Local hyperimmune response in lung tissue may be relevant in dexamethasone’s efficacy. However, a recent study showed that cytokine response to viral infection in human lung tissue is lower in SARS-COV-2 infection than SARS-CoV [31]. Alternative criteria, such as poor oxygen saturation levels, are also used as a rationale for dexamethasone use. Some evidence supports improvement in oxygen saturation by dexamethasone in COVID-19 patients [32]. Given the pleiotropic effects of steroids, yet unidentified mechanisms independent of immunosuppression or oxygen saturation may be responsible for the beneficial effects of dexamethasone.

Limitations of this study include retrospective experimental design, relatively low sample size and single-institution setting. In addition, our study population is limited to hospitalized patients. An inherent limitation of studying pathophysiological responses to human infection is that the accurate infection stage is difficult to assess, even with the date of positive laboratory findings. We used quantitative antibody responses to overcome this limitation. Cytokine concentrations were relatively stable throughout the two-week observation period and were similar to those reported in SARS-1. Suppression of exaggerated cytokine response by dexamethasone use was not observed, and only a very modest cytokine increase was observed even during the early stages of COVID-19 infection. It is reasonable to conclude that elevated cytokine responses in the “cytokine storm” range do not occur in COVID-19 patients. Thus, dexamethasone use appears to be neither required nor effective to control exaggerated systemic inflammation in COVID-19. This information can help make rational therapeutic choices and establish future guidelines for tocilizumab and dexamethasone use in COVID-19 patients.

## Figures and Tables

**Table 1:** Patient characteristics.

Parameter	No -DEX	DEX	*p* Value
**Sex, N (%)**			
Male	12 (71)	14 (74)	>0.99
Female	5 (29)	5 (26)	
**Age, mean (SD)**	62.1 (21.6)	71.8 (10.8)	0.091
**BMI, mean (SD)**	29.1 (7.3)	33.4 (6.9)	0.083
**Comorbidities, N (%)**			
Diabetes	9 (53)	10 (53)	>0.99
Hypertension	9 (53)	18 (95)	0.006
Obesity	6 (38)	13 (68)	0.095
**Coinfections, N (%)**			
Bacterial pneumonia	2 (12)	6 (32)	0.236
Fungal pneumonia	0 (0)	1 (5)	>0.99
Bacteremia	2 (12)	6 (32)	0.236
Urinary tract infection	2 (12)	2 (11)	>0.99
**Intubation, N (%)**	4 (24)	11 (58)	0.049
**ARDS, N (%)**	6 (35.3)	14 (73.7)	0.043
**Death, N (%)**	2 (12)	10 (53)	0.014

**Table 2:** Cytokine concentrations in COVID-19, influenza (CS), burn and control patients.

Cytokine	Influenza (CS)	Burn	No-DEX	DEX	Control
IL-6	5076 (2899, 8888)	3697 (1462, 9348)	52 ^a,b^ (13, 87)	63 ^a,b^ (33, 119)	24 (12, 48)
IL-8	557 (233, 1333)	199 (113, 349)	22 ^a,b^ (17, 27)	20 ^a,b^ (15, 26)	10 (7, 15)
TNF-α	414 (254, 672)	228 (171, 305)	52 ^a,b,c^ (35, 77)	68 ^a,b,c^ (52, 88)	26 (21, 34)
MCP-1	9732 (6728, 14,078)	2094 (1437, 3052)	971 ^a,c^ (745, 1265)	976 ^a,c^ (763, 1249)	306 (226, 416)
IP-10	124,434 (109 K, 140 K)	17,866 (13 K, 23 K)	2239 ^a,b,c^ (1014, 4947)	5885 ^a,c^ (3803, 9109)	238 (145, 389)

Geometric means of concentrations are shown in pg/mL with lower and upper 95% confidence limits in parenthesis. None of the cytokine concentrations were statistically different between the DEX and no-DEX groups (*p* > 0.01). N values are 17, 19, 9, 6 and 26 for the no-DEX, DEX, influenza (CS), burn and control groups, respectively. K represents ×1000. ^a^ *p* < 0.01 compared to influenza (CS) group. ^b^ *p* < 0.01 compared to burn group. ^c^ *p* < 0.01 compared to control group.

## Data Availability

Data is contained within the figures and tables of the article and Supplementary Materials.

## Supplementary Materials

The following are available online at https://www.mdpi.com/article/10.3390/idr13020036/s1, Table S1: Additional cytokine/chemokine concentrations in COVID-19, influenza (CS), burn and control patients, Figure S1: RBD antibody development in COVID-19 patients during hospitalization, Figure S2: Time-course of cytokine response in early and late stage COVID-19 infections.
