# Supplementary material for: Absence of “Cytokine Storm” in Hospitalized COVID-19 Patients: A Retrospective Cohort Study
Source: Infect Dis Rep. 2021 Apr 19;13(2):377–87. doi: 10.3390/idr13020036 (PMC8167630; doi:10.3390/idr13020036)

Supplemental Table S1. Additional cytokine/chemokine concentrations in COVID-19, influenza (CS), burn and control patients.

Cytokine	Influenza (CS)	Burn	No-DEX	DEX	Control
IL-1α	43<br>(13, 143)	18<br>(12, 26)	12<br>(7, 22)	8 [a]<br>(5, 12)	11<br>(8, 16)
IL-1β	33<br>(11, 99)	19<br>(12, 30)	7 [a]<br>(4, 14)	5 [a]<br>(3, 8)	8<br>(6, 10)
IFN-γ	82<br>(46, 148)	33<br>(12, 88)	17 [a, c]<br>(8, 38)	9 [a, c]<br>(6, 14)	3<br>(2, 3)
IL-10	290<br>(149, 566)	402<br>(275, 588)	43 [a, b, c]<br>(19, 97)	67 [c]<br>(43, 106)	9<br>(5, 15)
G-CSF	900<br>(284, 2846)	3859<br>(1935, 7698)	124 [a, b, c]<br>(68, 226)	156 [a, b, c]<br>(91, 268)	10<br>(6, 19)
M-CSF	1519<br>(1269, 1818)	267<br>(139, 514)	352<br>(168, 741)	305<br>(196, 476)	108<br>(61, 192)
IL-18	479<br>(232, 988)	345<br>(257, 463)	49 [a, b]<br>(26, 93)	92 [a, c]<br>(68, 123)	21<br>(13, 35)
MIP-1α	194<br>(110, 344)	155<br>(141, 171)	31 [a]<br>(14, 71)	26 [a, b]<br>(14, 51)	14<br>(11, 17)
MIG	44507<br>(32K, 62K)	52693<br>(42K, 66K)	5120 [a, b, c]<br>(3427, 7650)	7942 [a, b, c]<br>(6055, 10K)	2042<br>(1438, 2900)
MCP-3	562<br>(350, 902)	124<br>(97, 158)	34 [a]<br>(22, 52)	39 [a]<br>(26, 57)	26<br>(16, 43)

Geometric means of concentrations are shown in pg/ml with lower and upper 95% confidence limits in parenthesis. None of the cytokine concentrations were statistically different between DEX and No-DEX groups ($P > 0.01$). N values are 17, 19, 9, 6 and 26 for No-DEX, DEX, influenza (CS), burn and control groups respectively. K represents X1000.

a $P < 0.01$ compared to influenza (CS) group

b $P < 0.01$ compared to burn group

c $P < 0.01$ compared to control group

Supplemental Figure S1

RBD antibody development in COVID-19 patients during hospitalization.

Relative RBD IgG concentrations in No-DEX and DEX groups are shown across different days of hospitalization. In DEX group data points in blue represent days of dexamethasone use. Dashed line represents the upper 95% CI limits of antibody concentration in non-COVID-19 samples (223 RFU). Manufacturer suggested threshold value for a positive identification is 700 RFU. N = 17 for No-DEX and 19 for DEX groups on day 1.

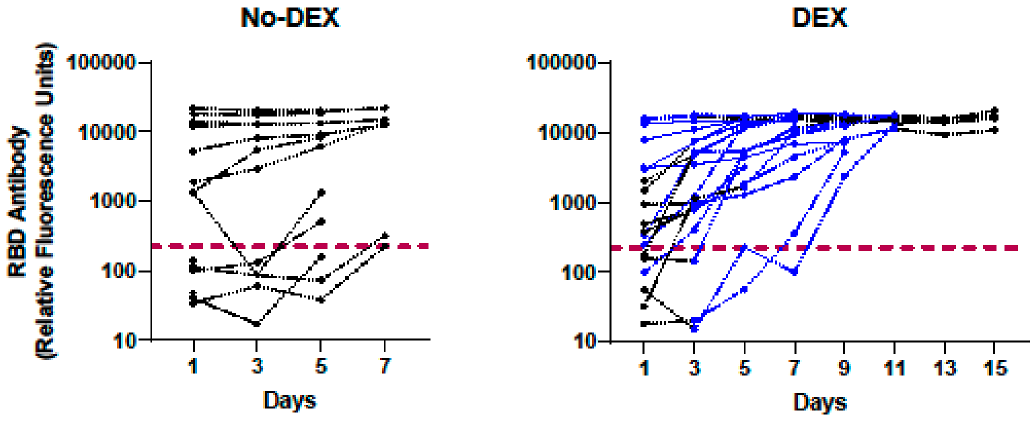

Supplemental Figure S2

Time-course of cytokine response in early and late stage COVID-19 infections.

Cytokine values are shown as geometric mean with 95% CI. N = 20 for early and 16 for late groups on day 1.

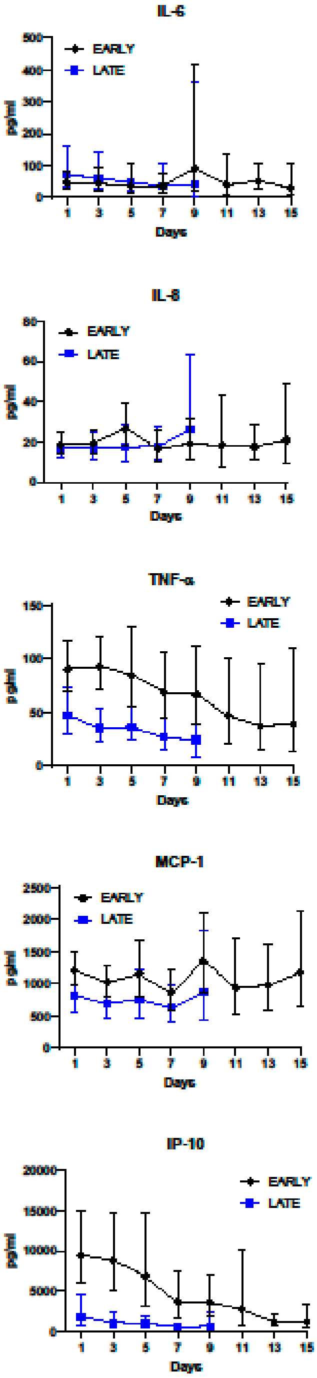

Supplement: Supplementary file 1 [file idr-13-00036-s001.zip › idr-1156543-supplementary.pdf]
